# Readmission of Patients with Acute Coronary Syndrome and Determinants

**DOI:** 10.5935/abc.20190104

**Published:** 2019-07

**Authors:** Larissa Marina Santana Mendonça de Oliveira, Ingrid Maria Novais Barros de Carvalho Costa, Danielle Góes da Silva, José Rodrigo Santos Santos Silva, José Augusto Soares Barreto-Filho, Marcos Antônio Almeida-Santos, Joselina Luzia Meneses Oliveira, Mirella Dornelas Batalha Moreira Buarque, Diva Aliete dos Santos Vieira, Antônio Carlos Sobral Sousa

**Affiliations:** 1 Universidade Federal de Sergipe - Departamento de Pós-Graduação em Ciências da Saúde, São Cristovão, SE - Brazil; 2 Instituto Federal de Educação Ciência e Tecnologia de Sergipe, São Cristovão, SE - Brazil; 3 Universidade Federal de Sergipe - Departamento de Nutrição, São Cristovão, SE - Brazil; 4 Universidade Federal de Sergipe - Departamento de Estatística, São Cristovão, SE - Brazil; 5 Universidade Federal de Sergipe - Departamento de Medicina, São Cristovão, SE - Brazil; 6 Fundação São Lucas - Centro de Ensino e Pesquisa, Aracaju, SE - Brazil; 7 Programa de Pós-graduação em Saúde e Ambiente da Universidade Tiradentes, Aracaju, SE - Brazil; 8 Hospital do Coração, Aracaju, SE - Brazil; 9 Fundação de Beneficência do Hospital Cirurgia, Aracaju, SE - Brazil; 10 Universidade Federal de Sergipe - Departamento de Nutrição, Lagarto, SE - Brazil

**Keywords:** Acute Coronary Syndrome, Hospitalization/economy, Patient Readmission/economy, Heart Failure, Delivery of Health Care/economy, Hospital Mortality, Socioeconomic Factors, Drug Therapy/economia

## Abstract

**Background:**

Acute coronary syndrome (ACS) is responsible for high rates of hospital admission and readmission, which are associated with increased costs for the patient and the health system, and increased in-hospital mortality rates.

**Objective:**

To evaluate readmission in patients with ACS and its determinants.

**Methods:**

This was a retrospective cohort study of adult and elderly patients with ACS, readmitted to public and private referral cardiology hospitals within one year after the first hospitalization for ACS. The occurrence of readmissions, the time elapsed from the first to the second admission, and the use of medications at admission were collected from the medical records. Associations between categorical variables were evaluated by the chi-square test or the Fisher's exact test. Multiple logistic regression was used to evaluate predictors for readmissions. A p < 0.05 was set as statistically significant.

**Results:**

Readmission rate was 21.5% (n = 115) and mean time between admissions was 122.7 ± 112.1 days. The patients were mostly men (64.0%), mean age of 63.15 ± 12.3 years. Among readmitted patients, 7% had a prognosis of "death", and 68.7% were readmitted more than once within a one-year period. The main reasons of readmission were cardiovascular diseases including ACS. Private health care and the diagnosis of congestive heart failure were associated with multiple logistic regression.

**Conclusion:**

ACS was the main cause of readmission, with higher prevalence among users of supplemental health care. Readmissions were associated with previous diagnosis of congestive heart failure and the type of health care provided.

## Introduction

During the last years, there has been increasing rates of hospital admission and readmission for ischemic heart diseases, including acute coronary syndrome (ACS).^1^ Hospital readmissions account for a significant increase in costs for the patient and public and private health system, and in hospital mortality. The risk of death in readmitted patients is greater when readmissions occur for cardiac causes.^2-4^

After an ACS event, hospital readmissions may be associated with different cardiac conditions, including myocardial ischemia, arterial fibrillation and uncontrolled hypertension, and also non-cardiac conditions such as poor treatment adherence, alcohol consumption, psychological factors, socioeconomical factors and health system issues.^5-7^ Besides, hospital readmissions are correlated with the quality of the health care provided and management of comorbidities during hospitalization, and hence may be used as quality indicators of the healthcare services.^8,9^ The type of health care system can also influence readmission rates, as it reflects different levels of access to health care and inequality between individuals.^8^

Therefore, the present study aimed to evaluate readmission rates after an ACS event among users of a public, supplemental health care system in the state of Sergipe, Brazil, and to evaluate the association between readmission rate and socioeconomic determinants of lifestyle and clinical outcomes.

## Methods

### Study design

This was a retrospective cohort study of patients with ACS, conducted between August 2017 and April 2018. A total of 581 patients was selected during the first admission (October 2013-September 2015) to one of the four referral hospitals in the city of Aracaju, Brazil. Three of these institutions provided private health services and one of them provided public services. All participants were evaluated at three time points throughout the study: at hospital admission, and 30 days and 180 days after the ACS event.^4^

Clinical diagnosis of ACS at admission and the type of ACS - acute ischemia, acute myocardial infarction with ST-segment elevation (STEMI), and acute myocardial infarction without ST-segment elevation (NSTEMI) - were collected from medical records. These diagnoses were made based on physician's interpretation of symptoms (consistent with acute ischemia in the 24 hours prior to admission) and/or on elevation of biomarkers of cardiac ischemia, confirmed by electrocardiography, Doppler echocardiography or coronary cineangiography.

Once personal data (name, date of birth, date of hospital admission) of patients were obtained, the medical records were searched for the occurrence of readmissions within one year after the first admission for ASC.

### Study sample

**Figure 1:**
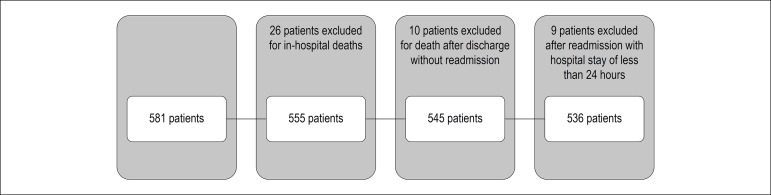
Flow chart of the occurrence of readmission within one year after the first admission in patients with acute coronary syndrome.

One-year readmission after first hospitalization for ACS was investigated in a cohort of 581 patients with ACS (Figure 1).

### Inclusion criteria

All patients admitted for ACS in cardiology referral hospitals in Aracaju, Sergipe, Brazil, identified at baseline of the study were included.

### Exclusion criteria

Patients who died during or after discharge of the first hospitalization, patients who were not readmitted after discharge of the first admission, and those who were presented readmission with less than 24 hours of hospital stay.

### Outcome measures

#### Socioeconomic status

Patients' personal data were retrieved from the database of first admitted ACS patients during the period from October 2013 to September 2015.^4^ A semi-structured questionnaire was used at the time of admission for ACS. Data of age (adults and elderly patients), sex (female and male), self-referred race (white, pardo, black), educational attainment (more or less 9 years of schooling), per capita income (less than 1 salary/person, 1-3 salaries/person, 3-5 salaries/person and more than 5 salaries/person), type of health care (public or private), life habits - alcohol consumption and smoking, reported by the patient or caregiver, and physical activity (classified by the international physical activity questionnaire, IPAQ) - nutritional status according to the body mass index (BMI) and waist circumference (WC). BMI was classified into low weight, normal weight and overweight, according to the World Health Organization (WHO)^10^ cut-off points (for adults) and the Pan American Health Organization (PAHO)^11^ cut-off points (for older patients). A WC < 80 cm for women and < 94 cm for men was considered normal.^10^

#### Hospital readmission

Occurrence of readmissions within one year after discharge was searched in the medical records of four cardiology referral hospitals. Data regarding the cause and date of readmissions and use of medications at the time of readmission were collected. All this information was transferred into a structured questionnaire.

Hospital admissions were categorized according to the primary diagnosis and the reason of admission - ACS, cardiovascular and noncardiovascular causes.

#### Drug therapy

After 30 and 180 days after discharge, the use, or not, of the following drugs was assessed: antiplatelet agents, statins, beta blockers, angiotensin-converting enzyme inhibitors (ACEI), and angiotensin receptor blockers (ARBs). The use of these medications at home was also evaluated at readmission (collected from the medical records).

#### Physical activity

The practice of physical activity was evaluated using the short version of the IPAQ.^12,13^ This instrument classifies the patient into four categories, based on the intensity levels of physical activity: very active, active, insufficiently active and inactive. In our study, "very active" and "active" individuals according to the IPAQ were considered "active", and those classified as "insufficiently active" and "inactive" in the IPAQ were considered "inactive". The level of physical activity at hospital admission, and 30 days and 180 days after discharge was assessed by telephone.

#### Clinical features

We evaluated the type of ACS at first admission, and the presence of previous systemic arterial hypertension (SAH), diabetes mellitus (DM), dyslipidemias, coronary artery disease (CAD), congestive heart failure, angina, AMI, angioplasty, myocardial revascularization and chronic kidney disease. All variables were categorized into present or absent (yes or no).

### Statistical analysis

Categorical variables were expressed as absolute and relative frequency, and distribution of quantitative variables was tested for normality using the Kolmogorov-Smirnov test. Variables that met the assumption of normality were presented as mean and standard deviation. Associations between categorical variables were assessed using the chi-square test or the Fisher's exact test, as appropriate. A p < 0.05 was set as statistically significant; all variables with p < 0.20 after association test and those that were risk factors for cardiovascular diseases were subjected to multiple logistic regression analysis.

Fourteen variables- sex, type of health care, race, age, hypertension, dyslipidemia, DM, congestive heart failure, use of statins on day 180, educational attainment, smoking, alcohol consumption, type of ACS - were selected as independent variables in the regression model, and readmission as dependent variable. The odds ratio (OR) and 95% confidence interval was used in the model. The Hosmer-Lemeshow goodness of fit test was used to evaluate the fitness of the model.

All analyses were performed using the R software, version 3.4.0 (The R Core Team, 2016).^14^

## Results

A total of 536 patients were considered eligible for the study. Mean age was 63.15 ± 12.26 years, and most of the were men (64.0%). One hundred fifteen readmitted patients were identified (21.5%). Readmissions occurred 122.74 ± 112.14 days after discharge from the first hospitalization. Among readmitted patients, 7% had the prognosis of "death", and 68.7% were readmitted more than once within a one-year period.

Regarding the reasons of hospital readmissions, 42.6% were ACS, 20.9% other cardiovascular causes (pulmonary hypertension, peripheral obstructive disease, second-degree atrioventricular block, and mitral insufficiency) and 36.52% other noncardiovascular causes (malnutrition, respiratory insufficiency, kidney disease, DM and complications, unspecified disorder of vestibular function, diverticulitis, hernioplasty, trauma and hyponatremia).

Table 1 presents the association of readmission with socioeconomic variables with the type of health care provided, nutritional status and life style. White patients, patients with higher educational attainment, those with higher family income and users of private health care services showed higher readmission rates.

**Table 1:** Socioeconomic data, nutritional status, lifestyle habits, and type of health care provided associated with readmission in patients with acute coronary syndrome in Aracaju, Brazil, 2017

Variable (%)	Readmission		P value*
	Yes	No	
**Socioeconomic variables**			
**Sex**			0.1201
Female	49 (25.39%)	144 (74.61%)	
Male	66 (19.24%)	277 (80.76%)	
**Race**			0.0008
White	55 (29.73%)	130 (70.27%)	
Black/pardo	54 (16.67%)	270 (83.33%)	
**Educational attainment**			0.0035
< 9 years	44 (16.18%)	228 (83.82%)	
≥ 9 years	71 (26.89%)	193 (73.11%)	
**Family income (per capita)**			0.0022
≤ 1 salary/person	12 (12.63%)	83 (87.37%)	
> 1 salary and ≤ 3 salaries/ person	20 (14.93%)	114 (85.07%)	
> 3 salaries e ≤ 5 salaries/ person	29 (23.97%)	92 (76.03%)	
> 5 salaries/ person	53 (29.12%)	129 (70.88%)	
**Age group**			0.0599
Adults	35 (16.99%)	171 (83.01%)	
Elderly	80 (24.24%)	250 (75.76%)	
**Nutritional status**			
**BMI**			0.8271
Low weight	6 (18.18%)	27 (81.82%)	
Normal weight	48 (22.43%)	166 (77.57%)	
Overweight	60 (20.91%)	227 (79.09%)	
**Abdominal circumference**			
Inadequate	83 (20.75%)	317 (79.25%)	1.0000
Adequate	27 (21.09%)	101 (78.91%)	
**Lifestyle habits**			
**Physical activity at admission**			
Inactive	66 (23.08%)	220 (76.92%)	0.3828
Active	49 (19.6%)	201 (80.4%)	
**Physical activity 30 days after admission**			
Inactive	89 (20.37%)	348 (79.63%)	0.6298
Active	11 (16.92%)	54 (83.08%)	
**Physical activity 180 days after admission**			
Inactive	62 (22.55%)	213 (77.45%)	0.2203
Active	36 (17.56%)	169 (82.44%)	
**Alcohol consumption**			1.0000
Yes	15 (22.06%)	53 (77.94%)	
No	100 (21.37%)	368 (78.63%)	
**Smoking**			0.6105
Yes	17 (18.89%)	73 (81.11%)	
No	98 (21.97%)	348 (78.03%)	
**Type of health care**			< 0.0001
Private	89 (29.47%)	213 (70.53%)	
Public	26 (11.11%)	208 (88.89%)	

* Chi-square test

Regarding clinical conditions associated with readmission (Table 2), readmission rates were higher in patients with STEMI, dyslipidemias, chronic kidney disease, congestive heart failure, angina, AMI, and previous angioplasty.

**Table 2:** Clinical conditions associated with readmissions in patients with acute coronary syndrome in Aracaju, Brazil, 2017

Variable	Readmission		P value*
	Yes	No	
**Type of ACS**			
Unstable angina	20 (20.83%)	76 (79.17%)	0.0205
NSTEMI	56 (27.45%)	148 (72.55%)	
STEMI	39 (16.53%)	197 (83.47%)	
Arterial hypertension	97 (22.99%)	325 (77.01%)	0.1255
Dyslipidemias	81 (27.93%)	209 (72.07%)	0.0001
Diabetes mellitus	48 (25.81%)	138 (74.19%)	0.0933
Previous CAD	59 (30.73%)	133 (69.27%)	0.0001
Congestive heart failure	39 (31.45%)	85 (68.55%)	0.0030
Angina	66 (25.38%)	194 (74.62%)	0.0408
Previous AMI	58 (30.53%)	132 (69.47%)	0.0002
Previous angioplasty	31 (32.29%)	65 (67.71%)	0.0066
Previous myocardial revascularization	12 (31.58%)	26 (68.42%)	0.1700
Previous chronic kidney disease	18 (51.43%)	17 (48.57%)	< 0.0001

* ACS: acute coronary syndrome; NSTEMI: acute myocardial infarction without ST-segment elevation (NSTEMI); STEMI: acute myocardial infarction with ST-segment elevation (STEMI); AMI: acute myocardial infarction; CAD: coronary artery disease; Chi-square test.

With respect to the use of medications after hospital discharge (Table 3), only absence of statin 180 days after the ACS event was associated with hospital readmission.

**Table 3:** Drug therapy associated with readmissions in patients with acute coronary syndrome in Aracaju, Brazil, 2017

Variable	Readmission		P value*
	Yes	No	
Without ASA for 30 days	3 (15.8%)	16 (84.2%)	1.0000
Without antiplatelet agents for 30 days	9 (30.0%)	21 (70%)	0.2111
Without statin for 30 days	6 (11.5%)	46 (88.5%)	0.2037
Without β-blockers for 30 days	12 (35.3%)	22 (64.7%)	0.0507
Without ACEI and/or ARBs for 30 days	6 (15.0%)	34 (85.0%)	0.4259
Without ASA for 180 days	5 (14.3%)	30 (85.7%)	0.4566
Without antiplatelet agents for 180 days	9 (16.4%)	46 (83.6%)	0.5640
Without statin for 180 days	8 (10.3%)	70 (89.7%)	0.0293
Without β-blockers for 180 days	12 (24.5%)	37 (75.5%)	0.6863
Without ACEI and/or ARBs for 180 days	16 (24.6%)	49 (75.4%)	0.5628

* acetylsalicylic acid (ASA); ACEI: angiotensin-converting enzyme inhibitors; ARBs: angiotensin receptor blockers; chi-square test or Fisher's exact test

Factors associated with readmission were subjected to multiple logistic regression analysis (Table 4). Type of health care and congestive heart failure showed statistical significance. Users of private health care services had higher odds of rehospitalization than those attending public health clinics. However, patients with previous congestive heart failure had 1.81 times greater odds of readmissions.

**Table 4:** Multiple logistic regression of variables in patients with acute coronary syndrome in Aracaju, Brazil, 2017

Variables	OR (95%CI)	P value
Male sex	0.78 (0.45; 1.38)	0.396
Public health care	0.46 (0.21; 0.98)	0.048
Elderly patients	1.11 (0.63; 1.99)	0.718
Black/pardo race	0.58 (0.34; 1.00)	0.050
Hypertension	1.72 (0.85; 3.73)	0.149
Dyslipidemia	1.69 (0.96; 3.04)	0.073
Diabetes mellitus	0.92 (0.53; 1.56)	0.749
Congestive heart failure	1.81 (1.01; 3.21)	0.042
Without statin for 180 days	0.76 (0.31; 1.70)	0.526
High school/higher education	1.56 (0.87; 2.85)	0.140
Smoking	1.43 (0.65; 2.99)	0.356
Alcohol consumption	1.93 (0.86; 4.22)	0.102
STEMI	1.30 (0.67; 2.61)	0.445
NSTEMI	0.97 (0.45; 2.13)	0.943

OR: Odds Ratio; STEMI: acute myocardial infarction with ST-segment elevation (STEMI); NSTEMI: acute myocardial infarction without ST-segment elevation (NSTEMI)

## Discussion

The readmission rate of 21.46% found in the present study was similar to that (24.5%) reported in the study by Dreyer et al.,^15^ who evaluated the rate of readmissions in 3,536 patients within one year after AMI in the USA. A study conducted in Canada with 3,411 patients of both sexes reported a higher readmission rate (61.7%) in one year after first hospitalization for ACS.^7^

Rehospitalization rates in our study were also lower than those reported by Ricci et al.(47.9%),^8^ evaluating 30-day readmission in a complex care hospital. Sangu et al.^16^ believe that readmission rates can be influenced by the time period of evaluation following discharge. Readmission rates can also be affected by geographic factors in a population.^17^

Readmission rate is also a predictor of the performance of hospital activities and the occurrence of complications after discharge,^18^ which can reflect the accessibility to primary health care services. Self-referral to hospitals may be explained by a difficult access to primary care services, and the idea of a treatment focused on acute rather than chronic care, which raises self-referral to complex care hospitals.^19,20^

Readmission rates, however, are not always associated with the access to health care centers and quality of care provided. The rates may be associated with complexity of disease and patients' individual features, including socioeconomic status and disease severity. On the other hand, planned readmissions have been associated with the quality of hospital care.^21,22^

The presence of other risk factors, particularly concomitant ones, increases the odds of readmission.^23^ The occurrence of comorbidities associated with ACS affects disease severity, and increases the need for invasive interventions. A study conducted by Belitardo & Ayoub,^1^ that evaluated readmission in elderly patients, reported that SAH, sedentary lifestyle, dyslipidemia, DM, stress, smoking and obesity increased the odds of requiring invasive procedures and readmissions.

Patient destination after discharge, which is related to disease severity, is also associated with 30-day readmission in patients with congestive heart failure, as discussed in the study by Mirkin et al.^24^ Patients had different destinations after discharge, and those requiring specialized support services had higher readmission rates.

The diversity of causes of readmission reinforces the importance of the access to regional health services and the degree of coverage of health services to reduce risk factors and complications that may affect readmission outcomes.^25^

Therefore, strategies aimed at reducing the occurrence of readmission should include planning of discharge, promotion of health education of patients and family members, scheduling of patients' follow-up before and after discharge, appropriate use of medications, effective communication between patients and healthcare staff at discharge and between the hospital and follow-up care.^26^

The present study showed that users of private health services showed higher odds of readmissions as compared with those attending public healthcare facilities. Also, the study showed an association between having access to private health services and the capacity of having health insurance. Thus, the type of health care is related to patients' socioeconomic status, race, educational attainment, family income and older age.^27^ In addition, the income may be associated with educational attainment, which indicates that people with higher education attainment have higher income and greater odds to have access to private health services. Therefore, users of health insurance plans have greater access to a high variety of health services,^2,28^ and an increase in the income is slightly associated with increasing acquisition of health insurance plans.

According to a Brazilian household budget survey (POF, Pesquisa de Orçamentos Familiares) conducted in 2008-2009,^29^ households spent an average of 5.9% of the family income with health care, including medications and health plans.^27,29^

A Brazilian nation-wide study by Malta et al.^27^ reported that in general, users of private health services are mostly women, white patients. Also, the use of health insurance plans is associated with socioeconomic status, reflected as race, educational attainment, family income and older age.

On the other hand, the public system is characterized by a difficult access to treatment, including invasive therapies such as myocardial reperfusion and medical treatment. Also, due to improvements in notification and classification of these events, complex cases are prioritized to hospitalizations.^30,31^ These findings corroborate previous results showing higher readmission rates in private health facilities than public ones.

The main reasons of readmission were cardiac conditions (53%), mainly ACS, which accounted for 42.6% of the total. The high prevalence of cardiac causes of readmission was also reported by Khawaja et al.^9^ (69%) evaluating 30-day readmission rates after percutaneous coronary intervention. Belitardo and Ayoub^1^ also found a higher frequency of ACS (41.6%) among readmissions.

First hospitalization and readmission may occur for the same diagnosis due to the lack of resolution of the primary cause, which characterizes an avoidable readmission.^1^ Avoidable readmissions are considered those that are the result of a situation that if managed differently might have prevented the admission.^32^

These findings were similar to those reported by Ricci et al.^8^ showing a higher prevalence of cardiac causes of readmissions. Besides, increased risk of readmissions due to cardiac causes after the first ACS event has been reported.^7^ In the study by Kwok et al.,^33^ although noncardiac causes were present in half of readmissions, cardiac causes were responsible for 46% of the cases, with chest pain of noncardiac origin and ACS the most prevalent diagnoses, respectively.

Different risk predictors of hospital readmission have been described in the literature. This may be explained by different characteristics of the study populations, including the presence of comorbidities, and particularities of health care centers.

Hospital readmissions may be related to several factors, such as patient's clinical condition, family and social support, and the quality of health care received during and after hospitalization. The high readmission rates draw attention to the need for the development of actions during hospitalization, at discharge, and during follow-up, considering patients' individual features, in order to reduce the number of readmissions.^20,34^

Our study has some limitations that should be considered. The lack of detailed information about the medications used by the patients and possible losses to follow-up. The readmissions were evaluated in the four main and referral cardiology hospitals of the city and therefore, a higher number of readmissions was somehow expected; we did not evaluate the occurrence of readmission in other hospitals in the city or in other cities. In addition, among these four institutions, there was only one public hospital, which requires referral for admission. These factors may have led to an underestimation of readmissions. On the other hand, this study design can be used to measure predictor variables before the occurrence of readmissions.

## Conclusion

ACS was the main cause of readmissions within one year after the first admission, with higher prevalence among users of supplemental health care. The main determinants of readmission were the type of health care provided, and a previous diagnosis of congestive heart failure. The present study adds to the knowledge on determinants of readmission and may serve as a basis for implementation of assistance strategies that may reduce the occurrence of new admissions in medium- and long term, leading to better prognosis and lower costs to the health system.
